# Ensemble and Single-Molecule Studies on Fluorescence Quenching in Transition Metal Bipyridine-Complexes

**DOI:** 10.1371/journal.pone.0058049

**Published:** 2013-03-04

**Authors:** Dominik Brox, Alexander Kiel, Svenja Johanna Wörner, Markus Pernpointner, Peter Comba, Bodo Martin, Dirk-Peter Herten

**Affiliations:** 1 Cellnetworks Cluster and Institute of Physical Chemistry, Heidelberg University, Heidelberg, Germany; 2 Institute of Physical Chemistry, Heidelberg University, Heidelberg, Germany; 3 Institute of Inorganic Chemistry, Heidelberg University, Heidelberg, Germany; Julius-Maximilians-University Würzburg, Germany

## Abstract

Beyond their use in analytical chemistry fluorescent probes continuously gain importance because of recent applications of single-molecule fluorescence spectroscopy to monitor elementary reaction steps. In this context, we characterized quenching of a fluorescent probe by different metal ions with fluorescence spectroscopy in the bulk and at the single-molecule level. We apply a quantitative model to explain deviations from existing standard models for fluorescence quenching. The model is based on a reversible transition from a bright to a dim state upon binding of the metal ion. We use the model to estimate the stability constants of complexes with different metal ions and the change of the relative quantum yield of different reporter dye labels. We found ensemble data to agree widely with results from single-molecule experiments. Our data indicates a mechanism involving close molecular contact of dye and quenching moiety which we also found in molecular dynamics simulations. We close the manuscript with a discussion of possible mechanisms based on Förster distances and electrochemical potentials which renders photo-induced electron transfer to be more likely than Förster resonance energy transfer.

## Introduction

Fluorescent probes are of general importance for analytical applications because of the high sensitivity reached by fluorescence detection. Fluorescent probes for sensing cations are of special interest not only to chemical or environmental applications but also for analytics in biology and medicine. [1], [2] Their importance is reflected in an almost exponential increase in publications when searching for 'fluorescent metal ion sensors' with to date about 1900 publications. [3] Their design principles and potential applications have been extensively reviewed over the last decade. [1], [2], [4], [5] The rational design of chemo-sensors links signaling to recognition by combining photo-physical changes with supra-molecular chemistry. Sensitivity and specificity of such probes are usually governed by the properties of the recognition moiety, the analyzed molecule and experimental conditions, like solvent, temperature, and pH. Signaling, on the other hand, is controlled by photo-physical processes linking the signaling moiety to the recognition part. Frequently applied mechanisms employ transfer of charge or energy as well as formation or disappearance of excimers or exciplexes. [1].

**Figure 1:**
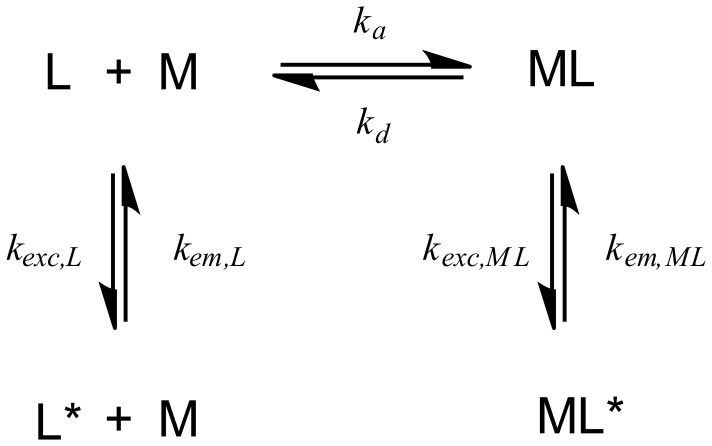
The fluorescent probe L can form a complex ML with the metal ion M yielding a second fluorescent species with reduced radiative rate (*k_em,ML_*).

Recently, we started to study the specific fluorescence detection of Cu^2+^ on the single-molecule level. We used a fluorescent probe that allowed us for the first time to measure complexation dynamics using single-molecule fluorescence spectroscopy (SMFS). [6] This probe consists of a bipyridine-derivative as the metal binding ligand and tetramethylrhodamine (TMR) as fluorescence reporter. Upon addition of CuSO_4_ we observe strong quenching with significant negative deviation from the usual linear Stern-Volmer law for bimolecular quenching. Because of their potential use for super-resolution localization microscopy, [7] we became interested in more detailed studies of suchlike probes. Knowledge about mechanism and kinetics of the quenching process are essential for designing new switchable probes for fluorescence microscopy. Therefore, we were interested in a quantitative description of the quenching process, in particular its negative deviation from Stern-Volmer law, and also in a variation of different fluorophores to extend the range of wavelengths. Among many alternatives, we found our observations best described by a model based on the formation of a less fluorescent complex. [8] Here, a quenched state with non-zero quantum yield is assumed (Fig. 1). The precise solution was introduced by Ryan and Weber in 1982 in the context of fluorescence quenching of fulvic acids with Cu^2+^and a simple approximation has been published by Warner et al. in 1986. [9], [10] We compared both the precise and the approximate model and used the simpler one to estimate stability constants and relative quantum yield of the quenched species from our data in this work (Fig. S1). We rule out energy transfer as possible quenching mechanism because the Förster distances determined for different metal ions are not reflected in our experimental data. However, we have strong indication that quantum yield in the copper complex is reduced by intramolecular electron transfer requiring conformations where complex and dye are very close to each other which correlates well with simulations from molecular modeling. At the same time the order of electrochemical potentials of the different metal ions is roughly reflected in our data.

## Materials and Methods

### Probe Preparation

Probe design was described in previous publications. [6], [11] Briefly, the probe (bipy-DNA) consists of a short double stranded DNA oligonucleotide (22mer) labeled with the chelating ligand 4,4′-dicarboxy-2,2′-bipyridine (bipy) on the 5′-end of one strand (5′-AAA AAC GCA AAG CAA GCG CGG G-3′) and a fluorophore on the 3′-end of the complementary strand (5′-CCC GCG CTT GCT TTG CGT TTT T-3′) such that bipy and the fluorophore are in close vicinity. The 5′-end of the dye-labeled strand is additionally modified with biotin to allow immobilization on glass cover slips for single-molecule experiments. The dye-modified oligonucleotides were prepared from 5′-NH_2_-modified oligonucleotides (IBA, Germany) by standard coupling reaction with N-hydroxysuccinimide (NHS) esters of ATTO620 and ATTO633 (ATTO-TEC, Germany). The TMR-labeled oligonucleotide was purchased from IBA. The dicarboxylic acid bipy was introduced by coupling the acid group to solid phase coupled DNA (MWG, Germany) (see [6] for detailed information).

### Ensemble Studies

Bulk studies were carried out by taking UV/VIS (Cary 500, Varian, Germany) and fluorescence spectra (Eclipse, Varian, Germany) in quartz glass cuvettes (45 µl, d = 3 mm; Hellma Analytics, Germany) at concentrations below 10^−4^ M in 10 mM MOPS buffer to avoid inner filter effects.

We studied the CuSO_4_-dependent quenching by measuring the change in relative quantum yield *φ_F_* instead of the fluorescence emission *F* to minimize errors (Fig. S2). For each CuSO_4_ concentration *φ_F_* was determined from three measurements at different probe concentrations to calculate *φ_0_/φ_F_* where *φ_0_* is the relative quantum yield of the sample containing no CuSO_4_ (Fig. S2). The quenching ratio *φ_0_/φ_F_* was then plotted against the CuSO_4_ concentration to study the quenching mechanism. Quenching was determined in the range of 0–20 µM CuSO_4_ where we observed saturation of the quenching without visible precipitation of CuSO_4_. Quenching by other metal ions was measured at the same probe concentration with similar concentrations of the respective salt (Fig. S3).

### Single-molecule Experiments

Single-molecule studies were carried out on a custom-built confocal microscope with two excitation lines at 635 and 532 nm and two detection channels at 570–605 nm and 650–720 nm. Excitation was achieved by a frequency doubled Nd:YAG laser (GL-30-A3, HB-Laser, Germany) at 532 nm and a pulsed laser diode (LDH-P-C-635, PicoQuant, Germany) at 635nm cycled at 80 MHz using a pulsed laser driver (PDL 800-B, Picoquant, Germany). The lasers were coupled into an inverted microscope (Zeiss Axiovert 100, Germany) and focused onto the sample with a 100× oil microscope lens (NA = 1.4, Olympus, Germany). Excitation was separated from fluorescence by a dichroic beam splitter (z532/633rpc, AHF Analysentechnik, Germany). Fluorescence emission was divided in the detection arm by another dichroic mirror (650DCXR, AHF Analysentechnik, Germany) and led through bandpass filters (680HQ65 for ATTO633, 650CLFP for ATTO620 and 585QM30 for TMR, Omega Optical, USA) for spectral separation. Fluorescence was detected by two avalanche photodiodes (APDs, AQR15H, Perkin Elmer, USA) and registered using a PC-board for time-correlated single-photon counting (SPC-134, Becker & Hickl, Germany). Custom-written software (LabView 7.1, National Instruments, USA) was used to synchronize data acquisition with sample scanning and positioning by a 3-axis piezo stage (P561.3CL, Physik Instumente, Germany), reaching 50 nm positioning accuracy.

### Molecular Modeling

Both fluorophore and bipy are coupled to DNA, which requires a complete conformational search and geometry optimization of the whole system including the DNA base pairs. The modeling was done with the program Maestro 9.2 (Macromodel v99109) [12] in combination with two different force fields, namely AMBER and OPLS_2005. [13], [14] The structural optimization proceeds through a conformational search, followed by a geometry optimization of a specific conformation which represents a local minimum. Due to the high dimensionality of the problem it cannot be expected that the global minimum is localized. During the conformational search valence angles and bond lengths are varied systematically until the resulting energy falls below a predefined threshold. In this study, the 'mixed torsional/low-mode sampling' approach in Macromodel was used. For a reasonable optimization it is helpful to fix specific geometric parameters defining, for example, the proximity of the fluorophore, i.e. TMR, and the bipy ligand or the planarity of TMR because a deviation from these constraints will lead to high energy geometries or to orientations of the fluorescing center and the complexing ligand, where the mutual influence is negligible and therefore is of no relevance for quenching processes. In our investigations we performed optimizations at first without the Cu^2+^-ion employing both force field parameterizations and secondly with a Cu^2+^-ion bound to bipy and complexed by two water molecules with the AMBER force field only. In the latter case we had to define a new atom type, representing the copper ion, as well as torsional and stretching interactions.

## Results and Discussion

**Figure 2:**
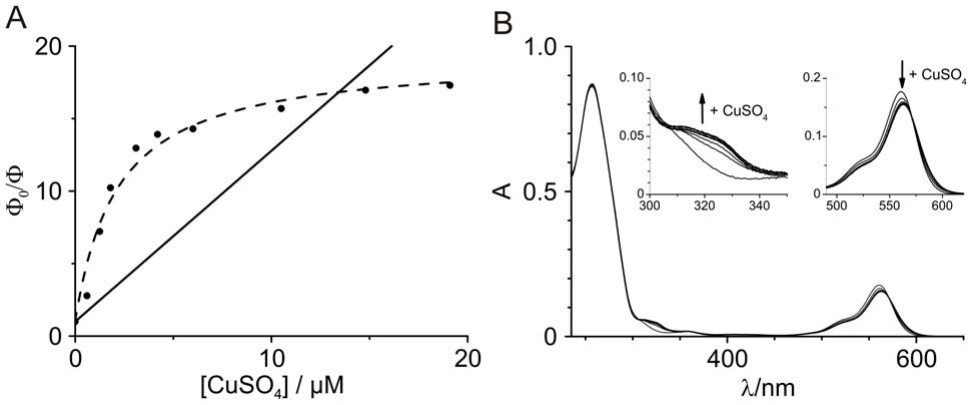
Fluorescence and UV/VIS studies on the quenching of TMR-bipy-DNA by CuSO_4_. (A) Stern-Volmer plot of TMR-bipy-DNA fluorescence quenching by CuSO_4_ (closed circles) shows a pronounced negative deviation from the Stern-Volmer law for collisional quenching (solid line) that can be explained using the model from eq. (1) for fitting the data (dashed line). (B) Absorption of TMR-bipy-DNA shows only a very weak dependency on the addition of CuSO_4_.

Quenching of fluorescence was measured in bulk experiments at different CuSO_4_ concentrations (0–20 µM). The quenching ratio *φ_0_/φ_F_* of the labeled probe plotted against the CuSO_4_ concentration (Fig. 2a, filled circles) shows a negative deviation from Stern-Volmer law (Fig. 2a, solid line). Considering the probe design, a negative curvature cannot be explained by the hindered access model which assumes multiple chromophoric groups. The strong quenching by Cu^2+^ at µM concentration is already indicating complexation. We also studied the effect of Cu^2+^ on the absorption of TMR-bipy-DNA (Fig. 2b) and found only small changes, i.e. a small increase of the absorption at 330 nm and a slight bathochromic shift by ∼2 nm at 551 nm accompanied by a small decrease in absorption. Although the absorption is notably influenced by addition of CuSO_4_ it cannot account for the observed decrease in fluorescence emission. The isosbestic point at ∼570 nm indicates the formation of a complex, which occurs at Cu^2+^-concentrations much larger than needed for fluorescence quenching. Thereby formation of a ground-state complex can be ruled out as main reason for the negative curvature (Fig. S4).

**Figure 3:**
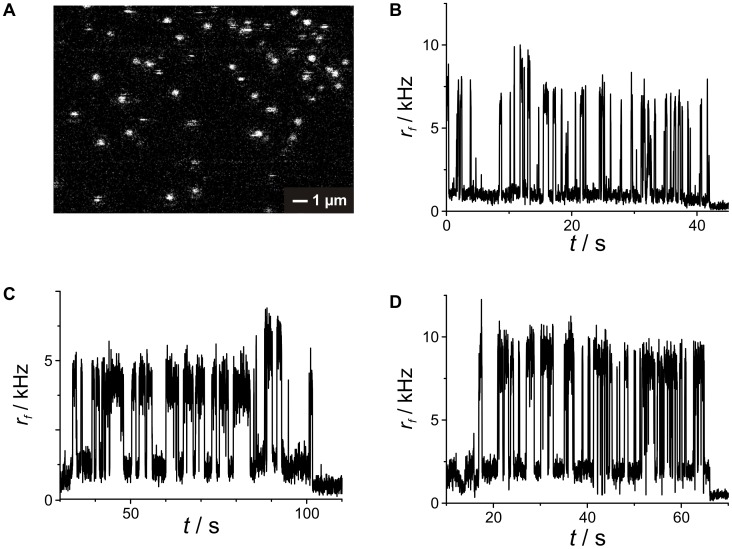
Single-molecule studies on the quenching of TMR-bipy-DNA by CuSO_4_. (A) Confocal fluorescence image of single ATTO620-bipy-DNA molecules immobilized on glass cover slides via biotin/streptavidin. Image (30×15 µm^2^) taken using pulsed excitation with a diode laser emitting at 635 nm with a repetition rate of 80 MHz at an average excitation power of 5.5 µW in presence of 0.1 µM CuSO_4_. Time-resolved traces of single immobilized dye-bipy-DNA molecules labeled with TMR (B), ATTO 620 (C) and ATTO 633 (D) recorded under the same conditions show discrete dark states due to reversible complexation of Cu^2+^ and bipyridine which leads to intramolecular quenching of the fluorescence emission.

We also studied the quenching in single-molecule experiments. Here, probes were immobilized on glass-covered slides using biotin-streptavidin binding described elsewhere. [6] The sample was imaged with a single-molecule sensitive confocal microscope to localize individual spots (Fig. 3a). The samples were excited with a pulsed diode laser emitting at 635 nm with a repetition rate of 80 MHz. To minimize photo-physical dynamics we applied an excitation power of 5.5 µW which is about a factor of 5–10 times below usual excitation power in confocal single-molecule fluorescence experiments. After addition of CuSO_4_ (0.1 µM), individual spots were brought into the confocal observation volume for time-resolved measurement of their fluorescence emission. Time-resolved data is shown in figure 3b, c and d for TMR-, ATTO620- and ATTO633-labeled bipy-DNA probes, respectively. The single-molecule data shows stochastic blinking of the probes with distinct bright states corresponding to the free ligand and dim states reflecting the quenched Cu^2+^ complex. Close inspection of the data shows that background intensity after photo-bleaching is significantly reduced compared to the dim states of all three probes. For comparison with bulk data, intensities were accumulated in histograms to identify the brightness of the different states (see Fig. S5 for an example). For each sample we evaluated 10–20 traces to obtain a trend for the relative quantum yield of the dim state *f_PQ,sm_* by dividing its brightness by that of the bright state after correcting for background. The estimates of the relative quantum yield of the dim states *f_PQ,sm_* are shown in table 1. They give a strong indication that the usual assumption of the quenched state showing no emission is wrong for the compounds used in this work. Possible mechanisms explaining this behavior include photo-induced electron transfer or resonance energy transfer. Both mechanisms depend on the distance between quenching moiety and dye and should be highly efficient if the two are in close contact. The linkers used to couple TMR and bipy to DNA introduce many degrees of freedom to the conformational space of both moieties. This could lead to residual fluorescence emission because of distance-dependent quenching efficiencies of the Cu^2+^/bipy-TMR system in different conformations. Models describing this behavior are present in literature. [9], [10] They assume an equilibrium between the free probe and the probe-complex (Fig. 1) in which the inverse fraction of residual fluorescence *F*_0_/*F* depends on the quencher concentration *c*(*Q*), equilibrium constant *K* of the complex formation and the relative quantum yield *f_PQ_* of the Cu^2+^/TMR-bipy-DNA complex (Eq. 1).
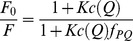
(1)


**Table 1:** Model parameters estimated by using eq. (1) to fit data for fluorescence quenching of different dye-bipy-DNA by Cu^2+^.

dye label	log *K* a	*f_PQ, bulk_* b	χ^2^	*f_PQ,sm_* c
TMR	*6.95±0.05*	*0.05±0.002*	0.9256	*0.11±0.07*
ATTO620	*6.53±0.08*	*0.37±0.01*	0.9552	*0.17±0.08*
ATTO633	*6.01±0.07*	*0.17±0.01*	1.1474	*0.18±0.07*

a Stability constant for probe/Cu(II) complex and.

b quantum yield of probe/metal cation complex as determined from bulk data.

c Quantum yield as determined from single-molecule experiments from 10–20 sample traces each.

A fit of equation (1) to our data is shown in figure 2a (dashed line) yielding the parameters presented in table 1. Interestingly, the mathematical solution of this model is equivalent to a model involving excited state reactions, leading to a different fluorescent species. [15] However, excited state reactions can be ruled out here because complexation kinetics have been shown to be independent on excitation intensities. [7].

For comparison, we tested different dye labels, i.e. ATTO620 and ATTO633, for their susceptibility to quenching by Cu^2+^ in order to analyze the influence of the dye label in bulk and single-molecule experiments. Bulk data was generated by measuring the attenuation of fluorescence emission upon addition of increasing amounts of CuSO_4_ up to a concentration of 20 µM (see Materials and Methods). The inverse fraction of residual fluorescence was plotted against the CuSO_4_ concentration and equation (1) was fitted to the data to extract the stability constant log *K* and residual quantum yield *f_PQ,bulk_* (table 1). Stability constants of TMR- and ATTO620-labeled probes correlate quite well and show no significant influence on the Cu^2+^ complex formation. In contrast, ATTO633 seems to have a weak destabilizing effect on the Cu^2+^ complex as saturation is reached at higher concentrations of CuSO_4_. Bulk and single-molecule data show a similar trend with respect to the residual quantum yield *f_PQ_*. However, there is a difference in extent of the relative quantum yield of the quenched species for TMR and ATTO620 which might be explained by the low number of traces from single-molecule experiments. In addition, one has to keep in mind that the accuracy of the estimated stability for both methods is limited by the probe purity which depends on complete hybridization and equimolar mixing of the oligonucleotides. Nevertheless, the dim states observed in single-molecule experiments motivated the use of equation (1) modeling a quenched state with residual fluorescence emission. Still the question arises by which mechanism the residual emission can be explained.

To further study the ideas of different conformations possibly involved in fluorescence quenching, preliminary Molecular Dynamics calculations have been done on the TMR-bipy-DNA to find conformers for which the Cu^2+^/bipy complex is in close vicinity to TMR, and structures where it is not. Short molecular dynamics runs were done on the structures with and without coordinated Cu^2+^ which was added *ad hoc* to the force field with a fixed square-pyramidal coordination sphere containing two equatorial and one axial water ligand.

It was interesting to see whether also in the absence of Cu^2+^ an advantageous orientation of the two interacting units can be observed. If this were the case, an added Cu^2+^-ion would smoothly insert into the TMR/bipy cavity and get stabilized by complexation. Since fluorescence is efficiently quenched as soon as the Cu^2+^/bipy complex is in close vicinity to TMR the corresponding structures should also be favored in the modeling process and represent local minima. In a first optimization cycle employing the OPLS_2005 force field two structural deficiencies were obtained, namely a non-planar xanthene system and a broken DNA base pair. In all structures where these deficiencies occurred, spatial proximity of the TMR and the bipy ligand could not be achieved. An appropriate redefinition of the participating atom types cured this problem leading to a planar xanthene system and a fully paired DNA strain. In the subsequent cycles additional constraints had to be introduced in order to fix both nitrogens of bipy at the same side of the molecule (torsional constraint) and to enforce vicinity of TMR/bipy (distance constraint). Application of these constraints yielded local minima of the system with a TMR/bipy distance of 3.4Å. Despite the comparatively low energy of this structure, steric factors can impede a further coordination of a Cu(H_2_O)_6_^2+^ ion due to the narrowness of the coordination site. A modeling under inclusion of Cu^2+^ was therefore undertaken as well (see below).

In order to compare different force fields with each other a structural investigation was also performed using the AMBER force field. Hereby the selected OPLS_2005 minimum structures were reinserted into the optimization cycle now employing the AMBER force field. The major result of this optimization is an enlarged coordination site for the complexation of the Cu^2+^-ion apt for fluorescence quenching. It has to be kept in mind that for some conformations additional hydrogen bonds can be formed which leads to some energetic biasing even if they are unsuitable for Cu^2+^ complexation. In general, the AMBER and the OPLS_2005 structures are similar to each other and yield minimum conformations which are suitable for a Cu^2+^ complexation in close vicinity to TMR.

**Figure 4:**
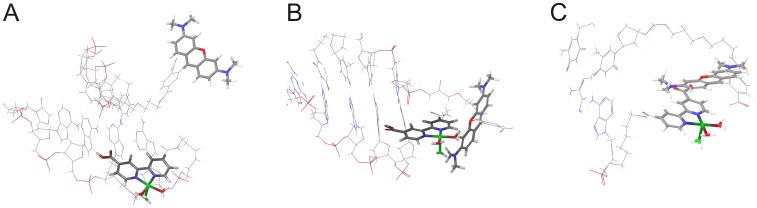
Energy optimized snapshots from different MD trajectories of Cu^2+^/TMR-bipy-DNA at large separation (A) and two different TMR/bipy orientations constrained at short separations (B, C).

The energetically most favorable structures were found to have a relatively large separation between TMR and the Cu^2+^/bipy (Fig. 4a). This might be due to the simplistic model (e.g. no explicit solvent, no exhaustive sampling) and has no chemical significance. When the structures were constrained to a closer TMR/bipy distance, a number of favorable structures were also found where the Cu^2+^ is in close-enough proximity to allow for fluorescence quenching (Fig. 4b, c). We do not report relative energies or lifetimes (see above), as our main focus here was to generate suitable starting structures for successive Quantum mechanical calculations.

For the structure investigation including the Cu^2+^-ion the AMBER force field was used exclusively. It turned out that after Cu^2+^ complexation numerous conformational searches led to structures with very remote locations of TMR and the Cu^2+^/bipy site. Hereby the bipy was partially immobilized by formation of hydrogen bonds to the DNA fragment and the resulting structures are very unlikely for inducing an efficient fluorescence quenching. Introducing a few specific constraints, however, solved the problem of too-distant ligands and yielded structures with a Cu^2+^/TMR distance of 5Å possessing a comparable energy as the ones with the very distant ligands. In these structures the bipy and the xanthene plane exhibit a non-planar orientation toward each other that is stabilized by hydrogen bonds between a Cu^2+^ coordinated water molecule and the carboxy group of the xanthene system.

In summary, by molecular modeling suitable conformations could be localized where the xanthene and the Cu^2+^/bipy moieties are close to each other, allowing for an efficient fluorescence quenching which can now be studied by *ab initio* methods. Thereby, the models support the previously formulated assumption of a Cu^2+^/TMR-bipy-DNA complex with changed fluorescence properties, i.e. decreased quantum yield.

Equation (1) was therefore further used to fit similar data acquired with different metal ions which are compared with data from literature in table 2. [16] Most obviously, Cu^2+^ leads to the strongest quenching where the model predicts a relative quantum yield of 5% for the complex. The Ni^2+^ complex shows a relative quantum yield of about 20% while all the other metal ions reach only 50% or more. The stability constants estimated are quite close to similar compounds published in literature although they do not match precisely. Except for Fe^2+^ the estimated stability constants are below the published values. As we could not find a published stability constant for the Mn^2+^/bipyridine complex we estimated it to be less than the corresponding phenanthroline complex. An explanation might be that the properties of the bipyridine moiety change upon conjugation to the oligonucleotide via esterification of one of the two carboxylic functions. Equation (1) fits quite well to all of the experiments, yielding χ^2^ always close to 1.

**Table 2:** Model parameters estimated by using eq. (1) to fit data for fluorescence quenching of TMR-bipy-DNA by different metal cations M^2+^ compare with data for respective 4,4′-dicarboxybipyridine complexes from literature and calculated Förster radii for energy transfer between TMR and the M^2+^-bipy complex.

metalcation	log *K* a	*f_PQ_* b	χ^2^	log *K* c	*R*_0_/nm
Cu^2+^	6.95±0.05	0.05±0.002	0.9256	8	1.5
Ni^2+^	6.56±0.03	0.21±0.01	1.0387	7.1	1.0
Co^2+^	5.10±0.38	0.77±0.05	1.1118	6.0	1.4
Mn^2+^	4.57±0.10	0.58±0.04	1.0323	<5^d^	1.8
Fe^2+^ e	5.13±0.08	0.49±0.02	0.9734	4.3	2.3

a Stability constants and.

b quantum yield of M^2+^/TMR-bipy-DNA complexes.

c Stability constants of M^2+^/4,4′-dicarboxybipyridine complexes from literature.

d No data for Mn^2+^/4,4′-dicarboxybipyridine complex given, but smaller than for phenanthroline (log *K* = 5).

e The experiments with FeCl_2_ might contain traces of Fe^3+^ because oxidation was not intercepted.

The data acquired for different metal cations now allows investigating resonance energy transfer as possible quenching mechanism. Using UV/VIS spectra of the respective complexes and the dye TMR we determined the Förster radii for different metal ions (Table 2 and Fig. S6) and found that they do not correlate with the observed quenching efficiencies. According to the Förster radius Co^2+^ and Mn^2+^ would also be expected to show a strong quenching which is not reflected in the data. We also studied electrochemical data available in the literature (Table S1) and found that the order of the electrochemical potentials of the cations (Cu>Ni≈Co>Fe>Mn) correlates relatively good with the strength of the quenching (Cu>Ni>Fe≈Mn>Co) with the exception of Co. The order of electrochemical potentials can only be taken as crude estimate as it will be influenced by complex formation as exemplified for the different type of complexes we found in literature (Table S1). Still, the main trend is resembled in the order of electrochemical potentials which makes electron transfer more likely than energy transfer.

### Conclusions

Our results from ensemble and single-molecule studies on fluorescent metal ion probes are best described by a reversible quenching reaction involving transitions between two states of different fluorescence quantum yield. We used this model to estimate stability constants and quantum yield of different metal complexes with a dedicated TMR-bipy-DNA probe that was originally designed for single-molecule studies on the reversible complexation with Cu^2+^. Our results compare well with data on bipyridine complexes from literature when accounting for the modification of one of the two carboxylic functions. In MD-simulations we found conformations in which the fluorophore (TMR) and the complex (Cu^2+^/bipy) are within 5Å which renders either Förster resonance energy transfer or photo-induced electron transfer a plausible mechanism for fluorescence quenching. However, Förster resonance energy transfer can be ruled out as the determined Förster radii suggest efficient transfer for all metal ions studied which is not in accordance with our data while electron transfer is more likely according to electrochemical data from literature. Still, more experiments and quantum mechanical modeling are required to finally prove the mechanism. Our experiments show the versatility of the DNA-based probe for characterizing the influence of different metal ions and different fluorescent labels. In future experiments, we also plan systematic studies on the influence of the ligand on the quenching. Beyond that, such kind of probes can be used in single-molecule experiments, e.g. to study the complexation dynamics, similar to previously published work. [6] However, here, we focused on the relative quantum yield of the quenched state which we found in good agreement with our ensemble data.

In summary, we characterized the quenching of our DNA-based probe based on a model for reversible quenching reactions. Molecular simulations further support the notion of intramolecular electron transfer as quenching mechanism. We also demonstrated the suitability of the DNA-based probe for systematic characterization of different fluorescent labels and different metal ions which can be easily extended to a systematic variation of ligand effects that can be studied by ensemble and single-molecule spectroscopy.

## Supporting Information

Figure S1
**Comparison of the precise model (solid line) with the approximate model (dashed line) for typical parameter values (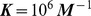 and 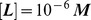).** The precise model can be found in Ryan et al. *Anal. Chem.* **1982**, *54*, 986–990. The simplified model (eq. 1) was taken from Patonay et al. *J. Phys. Chem.* **1986**, *90*, 1963–1966. A small difference between the two models arises only for 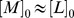 which is smaller than the experimental error. Therefore, the simpler model described by eq. (1) is used throughout the manuscript.(TIF)

Figure S2 **Relative fluorescence quantum yields determined from linear fits to fluorescence intensity plotted against maximum absorption.** The relative quantum yield *φ_F_* was determined at varying CuSO_4_ concentrations (0–20 µM) from the slope of a linear fit to the fluorescence intensity plotted against the absorption for three different probe concentrations. Validity of that approach was assumed as the respective absorption is not influenced by addition of CuSO_4_. The concentrations of the respective probes were 0.34, 0.47, and 0.68 µM for TMR-bipy-DNA, 0.37, 0.67, 0.82 µM for the ATTO620-bipy-DNA and 0.45, 0.56, 0.69 µM for the ATTO633-bipy-DNA, all in a range were re-absorption phenomena can be neglected.
(TIF)

Figure S3 **Stern-Volmer plot of TMR-bipy-DNA fluorescence quenching by different metal ions: (A) Ni^2+^; (B) Fe^2+^ (black), Co^2+^ (red) and Mn^2+^ (blue).**
(TIF)

Figure S4 **Determination of equilibrium constant** ***K*** **from the quotient of slope and y intercept of the linear fit to the data.** The plot shows slightly non-linear behavior for low concentrations of Cu^2+^. The equilibrium constant *K* was determined from the absorption spectra recorded at different concentrations of Cu^2+^. The spectra exhibit an isosbestic point at ∼570 nm indicating the presence of exactly two components. We used the Benesi-Hildebrand method to analyze the isosbestic point (Benesi et al. *J. Am. Chem. Soc.* **1949**, *71*, 2703–2707). The plot of (d[L]_0_[Cu^2+^])/(*A*–*A*_0_) against the concentration of Cu^2+^ shows almost linear behavior. Only for small concentration of Cu^2+^, the plot deviates slightly from linearity. A linear fit to the plot is used to calculate *K* as the quotient of the slope and the y intercept. This yields a value of log *K = *(5.16±0.07) M^−1^, which is about one order of magnitude smaller than the value determined from fluorescence quenching. This further supports our model of photo-induced electron transfer as quenching mechanism. It indicates the formation of another complex species at larger concentrations of Cu^2+^. The complex indicated in the UV/VIS spectra must therefore be different than the one observed in fluorescence quenching.
(TIF)

Figure S5 **Accumulated intensities of a typical trace of a single ATTO633-labeled bipy-DNA molecule (black).** Three Gaussian functions were fitted to the histogram (red) to estimate brightness of the different states (b: background; 1: dim state; 2: bright state). To determine the relative quantum yield of the complexed probes from single molecule experiments, the intensities of individual traces of single, immobilized probes were accumulated over time. Intensity histograms were fitted with multiple independent Gaussian functions to account for multiple peaks yielding an estimation of the average count rate of uncomplexed (2) and complexed state (1) as well as the background (b). For each trace the relative quantum yield of the complexed species was estimated from the ratio of the count rates of complexed and free state after background correction. The values of the relative quantum yield shown in the results section reflect the respective average and standard deviation over multiple traces.
(TIF)

Figure S6 **Overlap integrals** ***J*** **for TMR-bipy-DNA with different metal cations: Cu^2+^ (black), Ni^2+^ (blue), Co^2+^ (pink), Mn^2+^ (red), Fe^2+^ (green).** Förster radii for different metal ion complexes were calculated from absorbance and emission spectra to test if resonance energy transfer could explain fluorescence quenching for the TMR-bipy-DNA probe (Lakowicz, J.R.; Principles of Fluorescence Spectroscopy, 3^rd^ edition, Springer, New York, 2006). The donor emission spectrum was acquired from a 500 nM solution of the TMR-bipy-DNA in copper-free MOPS buffer (10 mM). The acceptor extinction spectrum was recorded from a 1∶1 mixture of the corresponding metal cations and a probe only carrying the ligand moiety in 10 mM MOPS buffer at a final concentration of 500 nM. For calculation of *R_0_* we used values of *Φ_0_ = 0.1, κ^2^* = 2/3 and *n* = 1.33 (Massey et al. *Anal. Chim. Acta* 2006, *568*, 181–189; Koerner et al. *Biophys. J.* 2011, *101*, 362–369). The results are shown in table 2 in the main manuscript.
(TIF)

Table S1 **Reduction potentials for different metal ions and complexes given in V.**
(PDF)
