# Supplementary figures and images for: Ensemble and Single-Molecule Studies on Fluorescence Quenching in Transition Metal Bipyridine-Complexes
Source: PLoS One. 2013 Mar 4;8(3):e58049. doi: 10.1371/journal.pone.0058049 (PMC3587577; doi:10.1371/journal.pone.0058049)

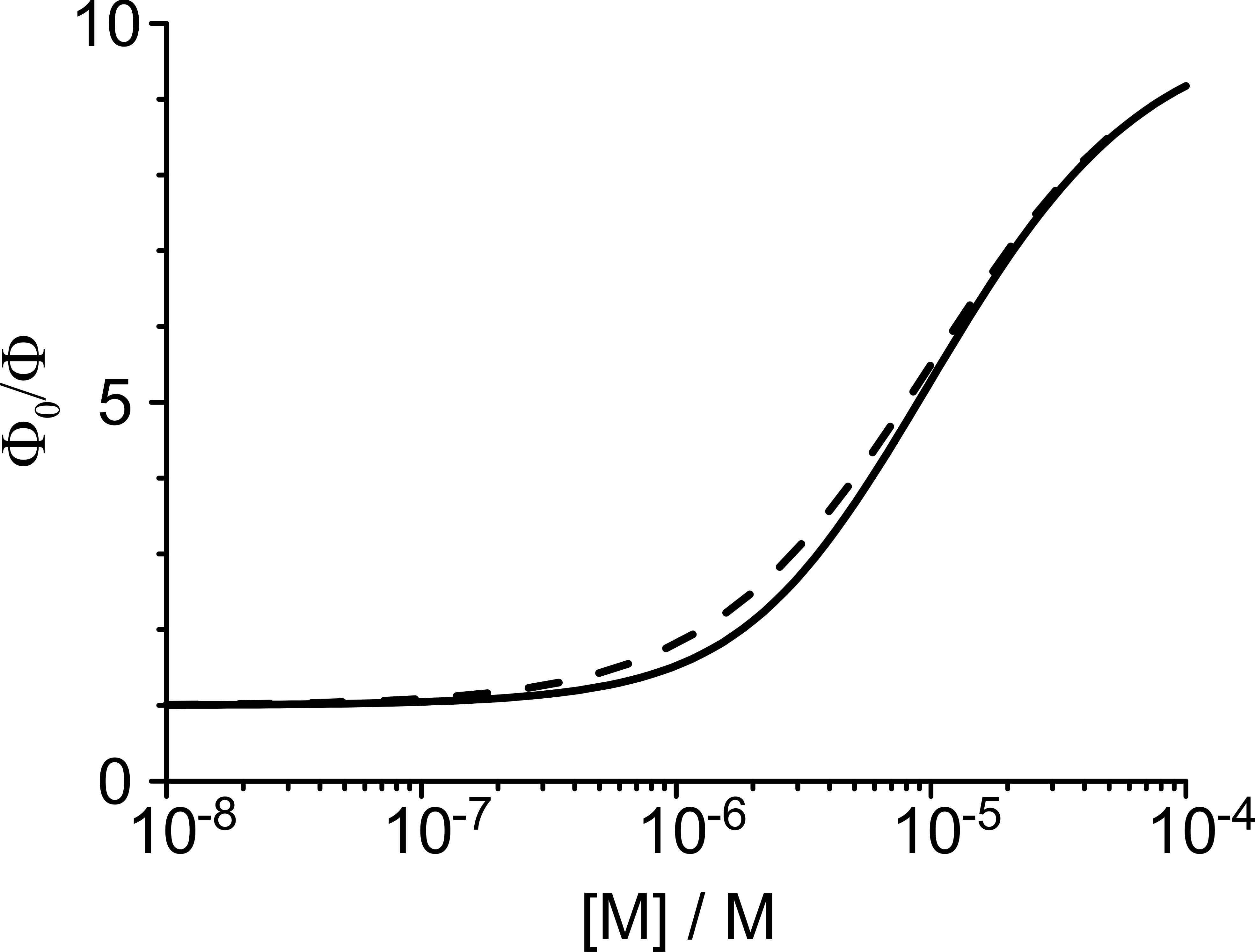

Supplement: Figure S1 — Comparison of the precise model (solid line) with the approximate model (dashed line) for typical parameter values ( and ). The precise model can be found in Ryan et al. Anal. Chem. 1982, 54, 986–990. The simplified model (eq. 1) was taken from Patonay et al. J. Phys. Chem. 1986, 90, 1963–1966. A small difference between the two models arises only for which is smaller than the experimental error. Therefore, the simpler model described by eq. (1) is used throughout the manuscript. (TIF) [file pone.0058049.s001.tif]
