# Supplementary material for: Ensemble and Single-Molecule Studies on Fluorescence Quenching in Transition Metal Bipyridine-Complexes
Source: PLoS One. 2013 Mar 4;8(3):e58049. doi: 10.1371/journal.pone.0058049 (PMC3587577; doi:10.1371/journal.pone.0058049)
Supplement: Table S1 — Reduction potentials for different metal ions and complexes given in V. (PDF) [file pone.0058049.s007.pdf]

Complex species	Cu^{2+}	Ni^{2+}	Co^{2+}	Fe^{2+}	Mn^{2+}
M^{2+} [a]	0.16	-0.27	-0.28	-0.44	-1.18
$M(bipy)^{2+}$ [b]	0.1	-	-	-	-
$M(bipy)_3^{2+}$ [c]	-	-1.26	-0.95	-1.35	-1.36

[a] Reduction potentials taken from http://www.webelements.com/ (accessed January 17, 2013), [b] measured against SCE, [c] reduction potentials taken from Richert et al. *Inorg. Chem.* **1989**, *28*, 2471-2475.
